# Intraoperative Epicardial Ultra-High Frequency Ultrasound in Coronary Artery Bypass Grafting Surgery

**DOI:** 10.7759/cureus.22649

**Published:** 2022-02-27

**Authors:** Ashley V Fritz, Archer K Martin, Erol Belli, Steven R Clendenen

**Affiliations:** 1 Anesthesiology and Perioperative Medicine, Mayo Clinic, Jacksonville, USA; 2 Cardiovascular Surgery, University of South Florida, Tampa, USA

**Keywords:** ultra-high frequency ultrasound, coronary arteries, cardioplegia, epicoronary, coronary artery plaque

## Abstract

The use of intraoperative epicardial ultrasound in order to aid physicians and surgeons in open cardiac surgery has been established for quite some time. Recently, the development of ultra-high frequency ultrasound (UFHUS), 50-70 megahertz (MHz) technology has resulted in high-resolution imaging capabilities previously unavailable for clinical use. This report is the first to describe the use of intraoperative UFHUS epicoronary scanning to assess coronary anatomy and visualize cardioplegia flow within native coronary vessels.

## Introduction

The use of intraoperative epicardial ultrasound to scan coronary arteries in order to assess bypass graft targets and survey efficacy of surgical re-vascularization is well described in the literature [1]. Whereas the international Registry for Quality Assessment with Ultrasound Imaging and Transit Time-flow Measurement (TTFM) in Cardiac Bypass Surgery (REQUEST) trial utilized a 15 MHz probe for scanning, recent literature describes the use of an ultra-high frequency ultrasound (UHFUS) 70 MHz probe for anatomical assessment during non-cardiac surgery [1-3]. We present a case of intraoperative UHFUS use for epicoronary scanning in a patient undergoing coronary artery bypass grafting (CABG) surgery.

## Case presentation

**Figure 1:**
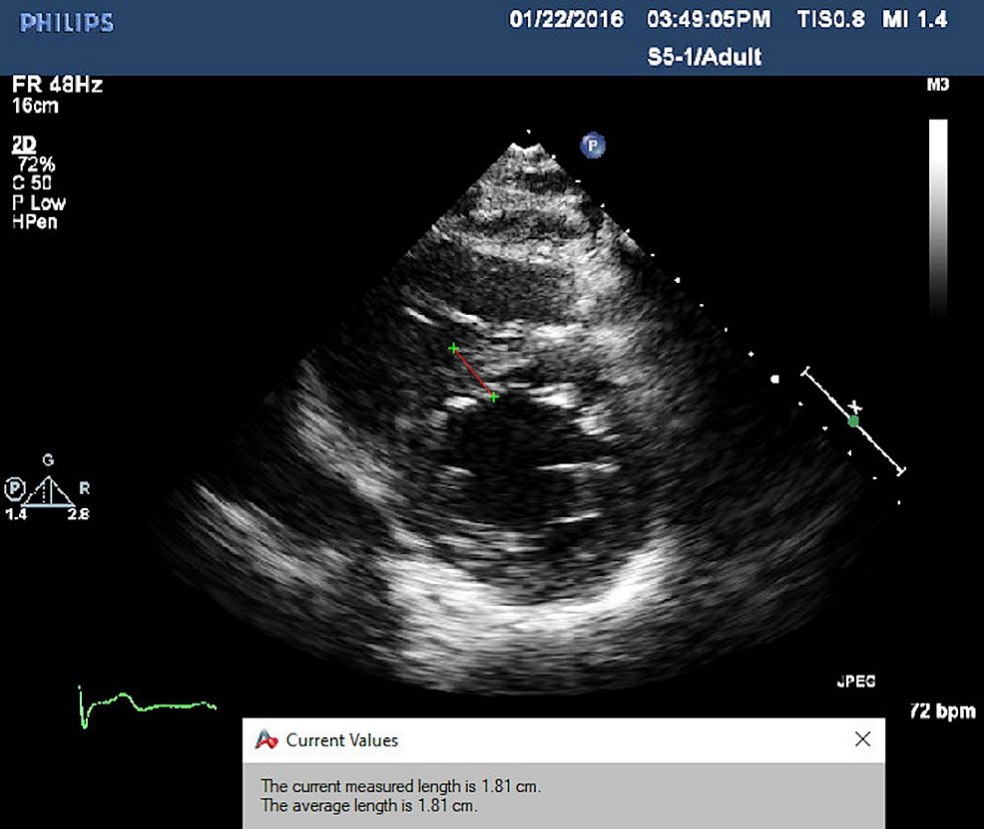
The parasternal short axis shows the concentric ventricular hypertrophy

A 64-year-old male with American Society of Anesthesiology (ASA) III status with stable angina was referred to our service for evaluation of his coronary artery disease. He had a past medical history significant for hypertension and hyperlipidemia. The patient had no prior cardiac surgical history and had successfully undergone laparoscopic Roux-en-Y gastric bypass surgery ten years ago. On physical exam, his vital signs were within normal limitations, his body mass index was 30, and he had no known allergies to medications. He reported worsening chest pain with activity over the year prior and was subsequently evaluated with both non-invasive and invasive cardiac testing. Transthoracic echocardiography showed concentric left ventricular hypertrophy with intact biventricular function (Figure 1).

Left heart catheterization showed extensive coronary disease, including 95% left main coronary occlusion, multiple discrete left anterior descending (LAD) coronary occlusions ranging from 50%-100%, and a 90% right coronary artery (RCA) lesion. The patient was scheduled for an on-pump CABG surgery. 

**Figure 2:**
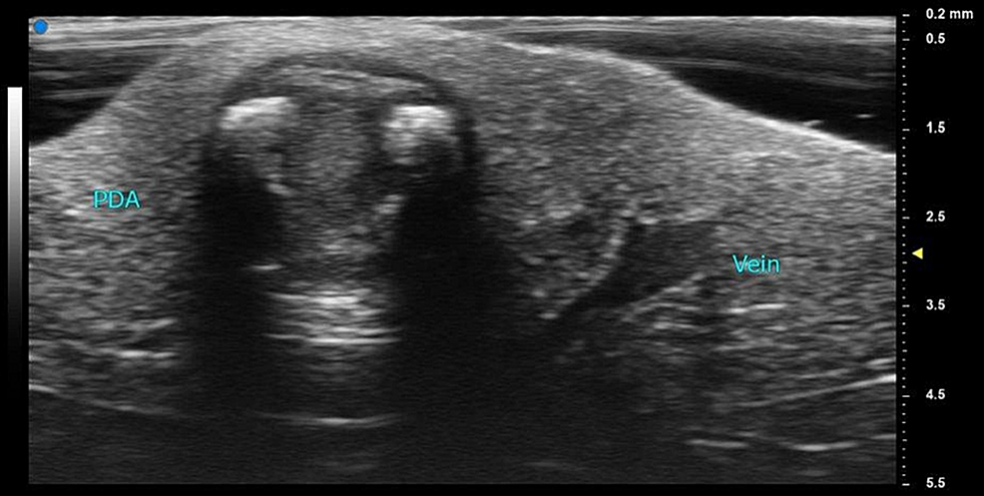
Short axis view of the posterior descending artery PDA - posterior descending artery

The patient was brought to the operating room, where he was placed under general anesthesia. After median sternotomy was performed, harvesting of saphenous vein and bilateral mammary arteries commenced without event. Both antegrade and retrograde cardioplegia were used to ensure electromechanical silence of the heart during cardiopulmonary bypass. A 70 MHz UHFUS probe was employed to assess the surface anatomy of the native, diseased coronary arteries in order to optimize anatomical planning for graft targets. After being placed in a sterile sleeve, the probe was used to assess the LAD and posterior descending artery (PDA; see Figure 2).

The significant calcifications and subsequent acoustic shadowing should be noted. A branch of the middle cardiac vein is present on the right side of the picture in Figure 2 and Video 1.

**Video 1:** This video shows the presence of retrograde cardioplegia flow within the cardiac arterial and venous system during cardiopulmonary bypass PDA - posterior descending artery

**Figure 3:**
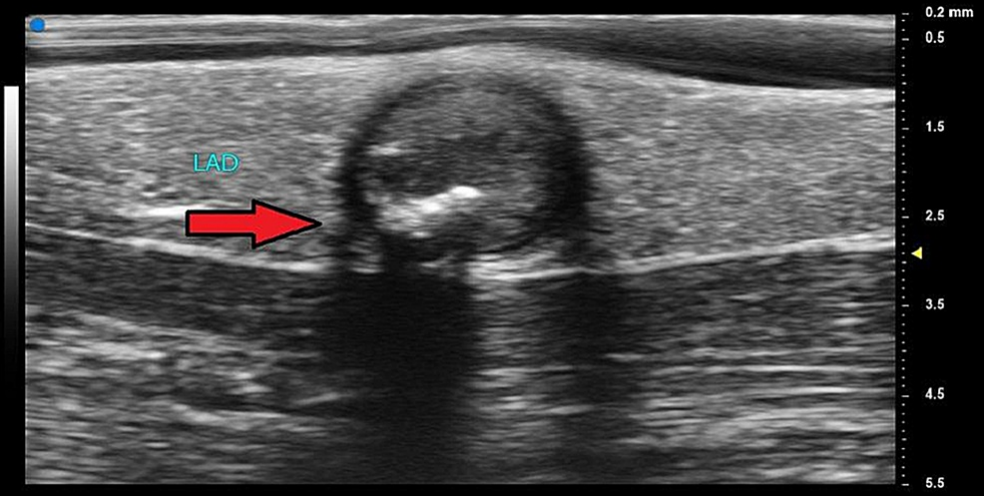
Short axis view of the left anterior descending artery The arrow shows the LAD in short axis, and calcifications are noted for both their bright echogenicity and ultrasonic dropout artifact. LAD - left anterior descending

Both cardioplegia flow and significant calcifications were seen in the PDA (Figure 3).

Coronary grafting commenced, and a Doppler flowmeter was used to confirm patency. The patient had a successful separation from cardiopulmonary bypass, an uneventful postoperative course, and was discharged on postoperative day seven. 

## Discussion

The REQUEST trial reported that the use of high-frequency ultrasound (HFUS) is not only helpful for evaluating coronary targets but that physicians should consider incorporating it into their usual practice for CABG surgery [1]. Higher frequency probes (55 Mhz) have been used to examine temporal arteries in the setting of giant cell disease, with greater resolution allowing for accurate assessment of intima layer thickness that correlated with histological diagnosis [3]. Recently reports have demonstrated that when used in conjunction with transit-time flow measurement, high-resolution epicardial ultrasound can minimize unnecessary graft revisions [4].

As the probe frequency increases, image resolution improves while sound penetration decreases [2]. Therefore, the use of UHFUS for epicoronary scanning presents a theoretical benefit over lower-frequency probes, yet no comparison of image quality was performed during this case. While our probe was rated up to 70 Mhz frequency, a frequency of 50 Mhz was used in order to achieve an appropriate ultrasound penetration depth of 5.5mm for epicoronary scanning. In our case, the UHFUS allowed for real-time evaluation of the extensive native coronary calcification during cardiopulmonary bypass. It can often be challenging to assess this in an arrested heart, and inadvertent selection of a distal graft site with heavy calcification requiring endarterectomy may lead to lower long-term patency. In addition to serving as an imaging aid for the selection of acceptable graft target sites, other potential applications of UHFUS epicoronary scanning include confirming cardioplegia flow in the setting of low coronary sinus pressures and appraising distal coronary flow post-revascularization. While its use allows for well-defined imaging, UHFUS is not without its disadvantages. In addition to the expense, the ability to maintain steady and optimized acoustic contact with the target vessel without distortion remains a concern [5].

## Conclusions

To our knowledge, this is the first report of the use of UHFUS for epicoronary scanning in CABG surgery. Further study is necessary to compare the efficacy of UHFUS versus high-frequency ultrasound for use in epicoronary scanning during CABG surgery. At the current time, all centers do not have immediate access to high-frequency ultrasound probes, and at present, there is no recommendation from the American Heart Association and the Society of Thoracic Surgery on the use of epicardial ultrasound scanning of coronary arteries, just for epiaortic scanning prior to cannulation. We hope that this report leads to further study of epicardial scanning for the societies to provide recommendations in the future.
